# Oxime Therapy for Brain AChE Reactivation and Neuroprotection after Organophosphate Poisoning

**DOI:** 10.3390/pharmaceutics14091950

**Published:** 2022-09-15

**Authors:** Darya A. Kuznetsova, Gulnara A. Gaynanova, Elmira A. Vasilieva, Rais V. Pavlov, Irina V. Zueva, Vasily M. Babaev, Denis M. Kuznetsov, Alexandra D. Voloshina, Konstantin A. Petrov, Lucia Y. Zakharova, Oleg G. Sinyashin

**Affiliations:** Arbuzov Institute of Organic and Physical Chemistry, FRC Kazan Scientific Center, Russian Academy of Sciences, Arbuzov Str. 8, 420088 Kazan, Russia

**Keywords:** cationic liposome, imidazolium surfactant, targeted drug delivery, acetylcholinesterase reactivation, blood-brain barrier

## Abstract

One of the main problems in the treatment of poisoning with organophosphorus (OPs) inhibitors of acetylcholinesterase (AChE) is low ability of existing reactivators of AChE that are used as antidotes to cross the blood-brain barrier (BBB). In this work, modified cationic liposomes were developed that can penetrate through the BBB and deliver the reactivator of AChE pralidoxime chloride (2-PAM) into the brain. Liposomes were obtained on the basis of phosphatidylcholine and imidazolium surfactants. To obtain the composition optimized in terms of charge, stability, and toxicity, the molar ratio of surfactant/lipid was varied. For the systems, physicochemical parameters, release profiles of the substrates (rhodamine B, 2-PAM), hemolytic activity and ability to cause hemagglutination were evaluated. Screening of liposome penetration through the BBB, analysis of 2-PAM pharmacokinetics, and in vivo AChE reactivation showed that modified liposomes readily pass into the brain and reactivate brain AChE in rats poisoned with paraoxon (POX) by 25%. For the first time, an assessment was made of the ability of imidazolium liposomes loaded with 2-PAM to reduce the death of neurons in the brains of mice. It was shown that intravenous administration of liposomal 2-PAM can significantly reduce POX-induced neuronal death in the hippocampus.

## 1. Introduction

Organophosphorus (OPs) compounds are a huge class of chemicals that, due to their unique properties and biological activity, are widely used in agriculture as pesticides [1], in medicine and veterinary medicine as anticancer, antiviral, and anthelmintic drugs [2,3], as well as in the production of lubricating fluids, plastics, and optoelectronic materials [4,5].

Moreover, some of them are prohibited chemical warfare agents [6,7,8]. The large number of poisonings with pesticides that are used in agriculture, in addition to the use of OPs in various terroristic attacks, has led to the search for and development of new effective approaches for the treatment of OPs poisoning [9,10]. The toxicity of OPs compounds is based on phosphorylation of the serine residue at the catalytic site of the enzyme acetylcholinesterase (AChE), which is responsible for the hydrolysis of the neurotransmitter acetylcholine (ACh). AChE deactivation leads to the accumulation of ACh in the cholinergic synapses of the central and peripheral nervous system, stimulating nicotinic and muscarinic cholinergic receptors [11,12]. In this regard, after poisoning, people develop an acute cholinergic crisis with excessive salivation, lacrimation, and bronchospasm, and neuromuscular blockade and death is possible [13,14]. Treatment of OPs poisoning currently involves three stages: (1) use of an anticholinergic drug (e.g., atropine), (2) use of quaternary oximes that reactivate cholinesterase (e.g., 2-PAM, obidoxime, HI-6), and (3) anticonvulsants (e.g., benzodiazepines) [15]. The main problem of quaternary oximes is their low ability to cross the blood-brain barrier (BBB) [16]. Therefore, quaternary oximes insignificantly reactivate phosphorylated AChE in the central nervous system (CNS) [16]. One of the ways to solve this problem is to create nanoparticles, loaded with oxime, that are able to cross the BBB [17,18,19,20,21].

Liposomes are widely studied as carriers of various types of drugs [22,23,24,25,26,27,28] and can potentially serve as carriers for drugs targeting the brain [29,30,31]. A large number of studies are aimed at creating liposomal dosage forms, as liposomes are biocompatible, biodegradable, and non-toxic [32]. The pathways for active transport of lipid nanocarriers through the BBB include receptor-mediated transcytosis, liquid-phase endocytosis, and adsorption-mediated transcytosis [33,34,35]. Because the endothelial cells that make up the BBB have many anionic sites on their surface, it is especially interesting to obtain cationic liposomes [36,37,38,39]. Therefore, the encapsulation of oximes in cationic liposomes may be a promising treatment for OPs induced poisoning.

**Figure 1:**
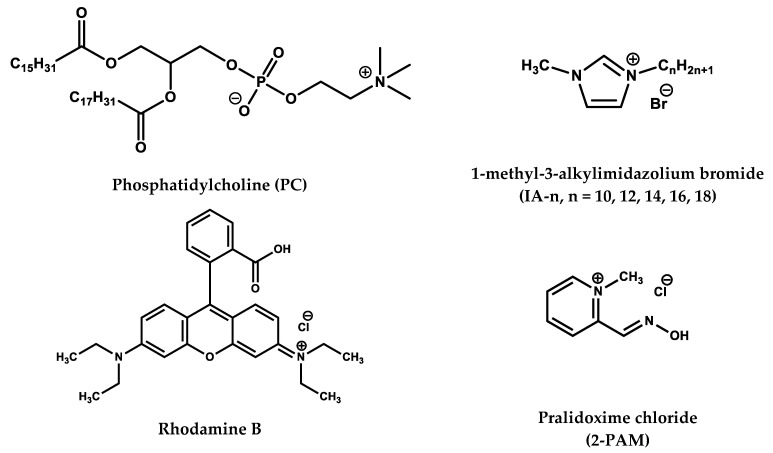
Chemical structure of compounds used in the work.

Thus, the goal of this study was to create new cationic liposomes loaded with 2-PAM to overcome the BBB, with a prolonged circulation time in the bloodstream and with a high value of brain AChE reactivation. The positive charge of liposomes can allow enhancement of the penetration of 2-PAM in the formulation into the CNS through the mechanism of absorption-mediated transcytosis [40,41,42,43]. Liposomes were non-covalently modified with cationic surfactants with an imidazolium head group and various length of the hydrocarbon chain. It is the first time that liposomes modified with imidazolium surfactants were used to pass through the BBB, which determines the novelty of the study along with the following tests on neuroprotection. The choice of imidazolium surfactants for the modification of liposomal systems on based the fact that imidazolium amphiphiles, due to the delocalized charge on the head group, can exhibit unusual properties (e.g., atypical mechanism of binding to the oligonucleotide, high antimicrobial activity, or formation of mitochondria targeting liposomes) [44,45,46,47,48]. In the first stage of the work, the main physicochemical properties of liposomes were assessed. The formed modified liposomes were loaded with a hydrophilic probes of rhodamine B or 2-PAM. For the systems, size, zeta potential, stability of particles over time, and in vitro release profiles were evaluated. For the first time, liposome penetration through the BBB and analysis of 2-PAM pharmacokinetics and brain AChE reactivation were studied in vivo. Importantly, an assessment was made of the ability of imidazolium liposomes loaded with 2-PAM to reduce the death of neurons in the brains of mice caused by POX poisoning. The structures of the compounds used in the work are shown in Figure 1.

## 2. Materials and Methods

### 2.1. Materials

Lipoid E PC (Phosphatidylcholine from egg) with a purity of 98% was a gift from Lipoid GmbH (Ludwigshafen, Germany). 1-methyl-3-alkylimidazolium bromide (IA-n, *n* = 10, 12, 14, 16, 18) was synthesized according to previously described procedures [49,50]. Rhodamine B (Sigma-Aldrich, St. Louis, MO, USA, ≥95%), and pyridine-2-aldoxime methochloride (2-PAM) (Sigma-Aldrich, St. Louis, MO, USA, ≥97%) were used for loading in liposomes. Purified water (18.2 MΩ cm resistivity at 25 °C) from Direct-Q 5 UV equipment (Millipore S.A.S., Molsheim, France) was used for the preparation of all solutions.

### 2.2. Preparation of Modified Liposomes

The preparation of modified liposomes was carried out by the method of hydration of a thin lipid film according to a previously developed technique [51,52,53]. Briefly: phospholipid and surfactant were taken in certain amounts and dissolved in ethanol (300 μL) to obtain a surfactant/lipid molar ratio of 0.02/1, 0.029/1, or 0.04/1. The alcohol was then evaporated on a rotary evaporator for 30 min at 40–45 °C under reduced pressure until the solvent was completely removed. The resulting fine lipid film was dispersed with water under stirring at a temperature of 60 °C for 1 h. The spontaneously formed hydrated lipid phase underwent five freeze/thaw cycles. The solution was then extruded 20 times through a 100 nm polycarbonate membrane (Whatman Nuclepore Track-Etched Membranes) using a LiposoFast Basic extruder (Avestin, Germany) to obtain unilamellar liposomes.

The preparation of liposomes loaded with substrates was carried out according to the same procedure, but the hydration of a thin lipid film was carried out with aqueous solutions of the substrates. The concentration of rhodamine B for loading into liposomes was 0.5 mg/mL, while for 2-PAM, it was 10 mg/mL or 20 mg/mL.

### 2.3. Dynamic and Electrophoretic Light Scattering

The physicochemical characteristics of the liposomes (hydrodynamic diameter D_H_, zeta potential ζ, polydispersity index PdI) were assessed by dynamic and electrophoretic light scattering on a ZetaSizer Nano device (Malvern Instruments Ltd., Malvern, United Kingdom). Measurements were performed at a liposome concentration of 1 mM. Calculation of the effective hydrodynamic radius (R) was performed by the Stokes-Einstein equation for spherical particles [54,55]: *D =* *kT*/6*πηR*, where *D* is diffusion coefficient, *k* is the Boltzmann’s constant, *T* is the absolute temperature, and *η* is the solvent viscosity.

The electrophoretic mobility data were converted by the original ZetaSizer software into the zeta potential according to the Smoluchowski equation [56]: *ζ = μη*/*ε*, where *ζ* is the zeta potential, *η* is the dynamic viscosity of the fluid, *μ* is the particle electrophoretic mobility, and *ε* is the dielectric constant.

All measurements were carried out at least three times.

### 2.4. Transmission Electron Microscopy

TEM images of liposomes were obtained in the Interdisciplinary Center for Analytical Microscopy of KFU, using a Hitachi HT7700 Exalens microscope (Tokyo, Japan). The images were acquired at an accelerating voltage of 100 kV. Before the study, 5 µL of the suspension was placed on a Formvar/carbon coated 3 mm copper grid, drying was performed at room temperature. After drying, the grid was placed in a transmission electron microscope [57].

### 2.5. Drug Release In Vitro and Quantitative Parameters of Encapsulation

The in vitro release rate of the substrates (rhodamine B, 2-PAM) from liposomes was assessed by dialysis. For the experiments, dialysis bags with a pore size of 3.5 kDa were used. Dialysis was performed in a phosphate buffer (pH = 7.4) at 37 °C. At certain time intervals, aliquots were taken from the buffer, and electronic absorption spectra were recorded on a Specord 250 Plus spectrophotometer (Analytik Jena, Jena, Germany). The optical density for rhodamine B was monitored at a wavelength of 555 nm, and for 2-PAM—at 294 nm. The extinction coefficients of the compounds were 94,000 M^−1^ cm^−1^ for rhodamine B, and 11,962 M^−1^ cm^−1^ for 2-PAM.

Spectrophotometry was also used to determine the encapsulation efficiency of the substrates. For this, 0.4 mL of a rhodamine B or 2-PAM solution was centrifuged in a centrifugal concentrator (Amicon Ultra-0.5, Merck Millipore, Darmstadt, Germany) for 10 min at 10,000 rpm. The concentration of substrates in the filtrate was than determined. Efficiency of encapsulation (EE) and loading (LC) were calculated using the following Equations (1) and (2) [58,59]:

(1) $$EE=\frac{Total\;amount\;of\;drug-free\;drug}{Total\;amount\;of\;drug}\times 100\%,$$

(2) $$LC=\frac{Total\;amount\;of\;drug-free\;drug}{Total\;amount\;of\;lipid}\times 100\%,$$

### 2.6. Hemolytic Activity and Hemagglutination Assay

The hemolytic activity of liposomal systems with encapsulated 2-PAM was assessed by the ability of liposomes to destroy erythrocytes. For this, the optical density of a solution containing liposomes was compared with the optical density of blood at 100% hemolysis. The object of the study was a 10% suspension of human erythrocytes. Hemolytic activity was assessed by the concentration of hemoglobin released into the plasma using an InVitroLogic microplate reader (Russia). A detailed description of the experiment can be found in [60].

Evaluation of the ability of liposomal systems to cause hemagglutination was carried out in a 96-well U-plate. For the experiment, a 2% suspension of erythrocytes was used. In short: a series of double dilutions of liposome solutions were prepared; 100 μL of liposomal solution was mixed with 100 μL of 2% erythrocyte solution and placed in a well. Each liposome concentration was placed simultaneously in two parallel wells. Then, incubation was carried out for 1 h at 37 °C. Hemagglutination was observed with the naked eye [17] and was assessed using a Nikon Eclipse Ci-S fluorescence microscope (Nikon, Tokyo, Japan) with a Ph1 condenser ring in phase contrast mode. A suspension of erythrocytes in 0.9% NaCl and a mixture of type A (II) and B (III) erythrocytes were used as negative and positive control of agglutination, respectively.

### 2.7. Animals

Experiments were carried out using Wistar rats (weighing 250–300 g, 12-weeks old) and CD-1 mice (weighing 25–30 g, 6-weeks old) of both sexes. The animals were purchased from the Laboratory Animal Breeding Facility (Branch of Shemyakin-Ovchinnikov Institute of Bioorganic Chemistry, Puschino, Moscow, Russia). The animals were kept in sawdust-lined plastic cages in a well-ventilated room at 20–22 °C in a 12-h light/dark cycle, with 60–70% relative humidity, and they were given ad libitum access to food and water.

### 2.8. Histology Analysis of Brain Sections

Free and encapsulated in liposomes Rhodamine B were injected into the tail vein of Wistar rats at a dose of 2.5 mg/kg. Untreated rats were used as a control for background fluorescence. Two hours after the injection, the animals were deeply anesthetized with isoflurane, and their brains were removed and frozen. The tissue was then cut into sections of 10 μm on a Tissue-Tek Cryo3 microtome (Sakura Finetek, Torrance, CA, USA). Images were observed using a Leica TSC SP5 MP confocal laser scanning microscope (Leica, Wetzlar, Germany), with an excitation wavelength of 550 nm and emission at 570 nm. 

### 2.9. Pharmacokinetic Study

For pharmacokinetic studies, the tail veins of Wistar rats were injected with liposomal 2-PAM at a dose of 25 mg/kg. At specified time intervals (5, 15, 30, 45, 60, and 120 min after injection), five rats were subjected to deep anesthesia (via inhalation of isoflurane) until the tail pinching reflex disappeared. To obtain plasma, blood was collected in 1 mL heparinized test tubes and centrifuged at 8000 rpm (8 min, 4 °C). After taking blood, rats were transcardially perfused with 300 mL of PBS (pH 7.4). Brain samples were then collected, and frozen in liquid nitrogen. The brain samples were weighed, and the total volume was brought to 10 mL with physiological saline (NaCl 0.9%) and homogenized.

For the analysis of 2-PAM in the samples, the method of high performance liquid chromatography with mass spectrometry detection (HPLC-ESI-MS) was used. Briefly: (1) 100 μL of plasma or 500 μL of brain homogenate was dosed into Eppendorf tubes (2 mL); (2) 200 μL (for plasma) or 1000 μL (for brain homogenate) of methanol was added to the samples, which were then mixed for 10 min at 5000 rpm on a vortex shaker (Neutec F202A0176 Model ZX3 Advanced Vortex Mixer, VELP Scientifica, Via Stazione, Usmate Velate MB, Italy); (3) to precipitate proteins, the mixture was centrifuged for 10 min at 10,000 rpm in a centrifuge (Eppendorf AG, Hamburg, Germany). The supernatant (methanol) was transferred to a beaker and the extraction procedure was repeated. Methanol was again transferred to a beaker and evaporated to dryness. The residue was then reconstituted with 1000 μL of methanol and centrifuged at 10,000 rpm for 5 min.

The supernatant was transferred into autosampler vials to prepare the samples for the chromatographic system (Agilent 1290 Infinity, Agilent Technologies Inc., Santa Clara, CA, USA), which was equipped with a pump, a vacuum degasser and an autosampler. Aliquots of 5 μL were run through at a flow rate of 0.3 mL/min using acetonitrile-0.1% formic acid in water (80:20, *v*/*v*) mixture as an isocratic mobile phase; the run time was 3 min. An Agilent 6550 iFunnel Q-TOF LC/MS (Agilent Technologies Inc., Santa Clara, CA, USA) mass-spectrometer with an electrospray ionization source in positive mode was used for quantitation with the following parameters: capillary voltage 3500 V, drying nitrogen gas 12 L/min (290 °C), and collision energy 20V. Detection of 2-PAM operating in the multiple reaction monitoring (MRM) mode was achieved with precursor–product ion transitions at *m*/*z* 137.0709→119.0603. The obtained data were processed with the Agilent MassHunter software (Agilent Technologies Inc.). Calibration was performed by plotting the 2-PAM peak integrals against the 2-PAM concentrations in the calibrating samples, which were prepared by adding 2-PAM to plasma or brain homogenate to obtain concentrations of 20, 50, 250, 500, 800, and 1500 ng/mL in plasma and 5, 50, 100, 150, and 200 ng/mL in brain homogenate and processing them through the extraction process. The calibration curves were linear in the range 20.0–1500.0 ng/mL for plasma and 5–200 ng/mL for brain homogenate and are provided in the supplementary data (Figure S1, Supplementary Materials).

### 2.10. Measurement of Brain AChE Inhibition and Reactivation Level

To evaluate the effectiveness of modified liposomes loaded with 2-PAM for brain AChE reactivation, a group of rats were poisoned with POX at a dose of 0.6 mg/kg, intraperitoneally. The control group was treated with an equivalent amount of saline. Brain samples for activity assessment were collected after 1 h. For trials for oxime treatment, 1 h after POX administration, two groups of rats were treated with free 2-PAM and 2-PAM encapsulated in IA-16/PC liposomes at a dose 25 mg/kg, intravenously. Following that, 3 h after liposome, or free 2-PAM administration (i.e., 4 h after poisoning), rats were anesthetized by isoflurane inhalation and transcardially perfused with 300 mL of PBS saline (pH 7.4). Brain samples were collected, frozen in liquid nitrogen, and stored at –80 °C. Brain samples were homogenized, and AChE activity was measured according to the method of Elman [61] as described by Dingova et al. [62]. Briefly, whole brain homogenates were prepared in a Potter homogenizer with 0.05 M Tris–HCl, 1% Triton X-100, 1 M NaCl, and 2 mM EDTA at pH 7.0 (1 volume of brain for 2 volumes of buffer) and 4 °C. The homogenates were centrifuged (10,000 rpm at 4 °C) for 10 min using an Eppendorf 5430R centrifuge with an FA-45-30-11 rotor (Eppendorf AG, Hamburg, Germany). For the AChE activity assay, 50 μL supernatant was incubated with 5 μL iso-OMPA of tetra-isopropyl pyrophosphoramide (iso-OMPA)—as a butyrylcholinesterase-specific irreversible inhibitor [63] at a final concentration of 0.05 mM for 30 min. The AChE-catalyzed hydrolysis reaction of substrate was then started by adding of 10 μL of acetylthiocholine iodide (final concentration 1 mM). After 10, 20, or 30 min of hydrolysis at 25 °C, reactions were stopped by adding the carbamylating agent neostigmine (0.1 mM). Samples were diluted in 50 mM phosphate buffer (pH 8.0), and DTNB (0.1 mM) was added. The amount of thiocholine produced during 20 min (10th–30th min) in homogenate samples was calculated. A sample without substrate was used as a blank. Three samples of each brain homogenate were measured independently. AChE activity was expressed in relation to the total amount of protein assessed by the Bradford method [64]. Each group of rats contained five animals. Inhibited AChE values were expressed as the mean ± the standard error of the mean.

### 2.11. Estimation the Level of POX-Induced Neurotoxicity

To assess the ability of liposomes loaded with 2-PAM to reduce the POX-induced death of neurons, the animal model of OPs-induced neurodegeneration was used [65,66]. CD-1 mice were divided into four groups (12 mice in each group): (1) a control group of intact mice; (2) a group of POX-poisoned mice that did not receive antidote therapy; (3) a group that received free 2-PAM (25 mg/kg, i.p.) and atropine (4 mg/kg, i.p.); and (4) a group of mice that received liposomal 2-PAM (25 mg/kg, i.p.) and atropine (4 mg/kg, i.p.).

After the administration of antidotes to the mice, 10 min later, the animals were injected subcutaneously with POX at a dose of 2 mg/kg. Four days after the administration of POX and antidotes, mice were anesthetized with isoflurane inhalation and transcardially perfused with 30 mL of PBS (pH = 7.4) and 30 mL of 4% paraformaldehyde (Panreac Química, Barcelona, Spain). The brain was fixed in 4% paraformaldehyde for 24 h at 4 °C before being placed in a 30% sucrose solution in PBS (pH = 7.4) with 0.01% sodium azide (Sigma-Aldrich, St. Louis, MO, USA) for 4 days. The brain was then frozen in a Tissue-Tek O.C.T. (Sakura Finetek USA, Inc., Torrance, CA, USA), and 25 μm sections were prepared using a Microm HM525 motorized cryostat (Thermo Scientific, Waltham, MA, USA). Staining with Fluoro-Jade B (FJB), a fluorescent marker of neuronal death, was performed as described in [67]. Briefly, the sections were placed on glass slides coated with 2% gelatin and dried at 50 °C for 30 min. The slides were then immersed in 1% sodium hydroxide dissolved in 80% ethanol for 5 min, then for 2 min in 70% ethanol before being washed for 2 min in distilled water. The glasses were then immersed in a 0.06% potassium permanganate solution for 10 min to reduce the background staining. The slides were rinsed with distilled water for 2 min and placed in a mixture of 0.04 mL of 1% FJB solution (Histo-Chem Inc., Jefferson, AR, USA) with 99.96 mL of 0.1% acetic acid solution for 20 min. The slides were then washed three times for 1 min in distilled water and dried at 50 °C for 10 min. Finally, the slides were washed in xylene for 5 min and covered with Immu-Mount (Sigma-Aldrich, St. Louis, MO, USA). Images were acquired using a Nikon H550S direct optical microscope with a Nikon digital camera. Analysis of neurons stained with FJB was performed using Image-J (NIH, Bethesda, MD, USA).

## 3. Results

### 3.1. Preparation and Characteristics of Empty Modified Liposomes

The production of cationic liposomes is a promising direction for creating nanocontainers capable of crossing the BBB and delivering drugs to the brain [40,68]. Therefore, we carried out the preparation of positively charged liposomes modified with cationic surfactants. To optimize the composition of liposomal systems, the preparation of empty lipid formulations was carried out by varying the surfactant/lipid molar ratios (0.02/1, 0.029/1, and 0.04/1). Evaluation of the hydrodynamic diameter (D_H_) of particles, their zeta potential, their polydispersity index, as well as their stability over time, was carried out by the methods of dynamic and electrophoretic light scattering (Table 1). The average D_H_ of liposomes was ~100 nm, the systems had a narrow size distribution (PdI did not exceed 0.25), and the zeta potential varied from +22 to +59 mV and depended on the molar ratio of the components and on the modifying amphiphile. In all cases, an increase in the zeta potential of liposomes was observed with an increase in the concentration of surfactants in the system. Modification of liposomes with cationic surfactants made it possible to significantly increase the stability of the systems. The mixed formulations were stable for 3–6 months, while the individual PC liposomes disintegrated after 3 weeks (Table 1). With the passage of time, the particle sizes remained practically unchanged; however, in some cases, a decrease in the zeta potential occurred, probably due to the release of amphiphilic molecules outside the lipid bilayer.

**Figure 2:**
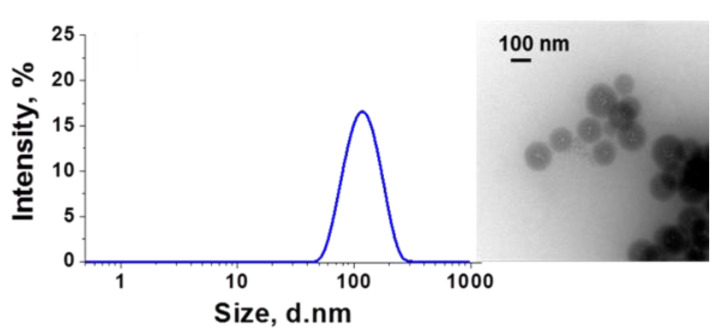
TEM and DLS images of the IA-16/PC modified liposomes with a molar ratio of 0.029/1, 25 °C.

To visualize the obtained particles, the method of transmission electron microscopy (TEM) was used (Figure 2). Micrographs were obtained for the IA-16/PC system at a surfactant/lipid molar ratio of 0.029/1. It can be seen that the sizes of the visualized liposomes completely coincide with the sizes obtained by the method of dynamic light scattering (~100 nm). The systems were highly monodisperse, and the liposomes had a uniform spherical morphology.

### 3.2. Preparation, Characteristics of Rhodamine B-Loaded Liposomes: In Vitro Release Study

The next stage of the work consisted of evaluating the physicochemical characteristics of the modified liposomes when loading a hydrophilic probe, rhodamine B. An evaluation of the obtained data shows that the sizes of liposomes containing rhodamine B were close to those of unloaded systems (Table 2); the zeta potential was slightly higher than that of empty formulations, and was in the range +39–+65 mV. Liposomal systems showed high stability (more than 2 months). In addition, for these systems, high values of the encapsulation efficiency (EE) were noted at the level of 67–81%. The highest EE value were observed for liposomes modified with higher homologues (IA-16/PC; IA-18/PC).

**Figure 3:**
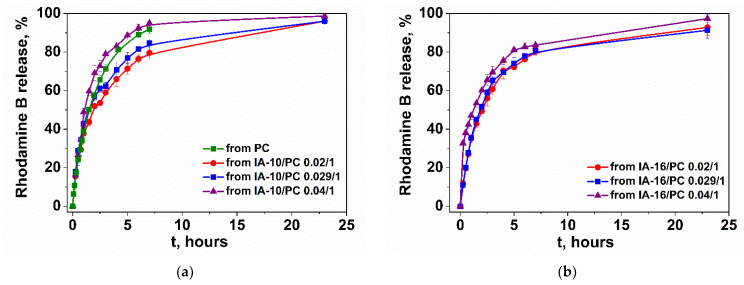
In vitro rhodamine B release from mixed liposomes at various surfactant/lipid molar ratios: (**a**) IA-10/PC; (**b**) IA-16/PC; phosphate buffer (0.025 M), pH 7.4, 37 °C.

To assess the release rate of rhodamine B from the modified liposomes, a dialysis technique was applied using a spectrophotometric method. For this, during dialysis, an aliquot of the external solution was taken at regular intervals and the absorption spectra of rhodamine B were recorded (Figures S2–S6). According to the results of spectrophotometric analysis, the dependences of the release of rhodamine B on time were plotted for different liposomal solutions (Figure 3 and Figure S7). Analysis of the data shows that rhodamine B is released from PC liposomes for about 4 h, and its encapsulation in modified liposomes allows an increase in the release time of the probe (Figure 3). During 9 h of dialysis, about 80% of the substrate was released from the modified liposomes.

### 3.3. BBB Penetration Capability of Imidazolium Surfactant Modified Liposomes

**Figure 4:**
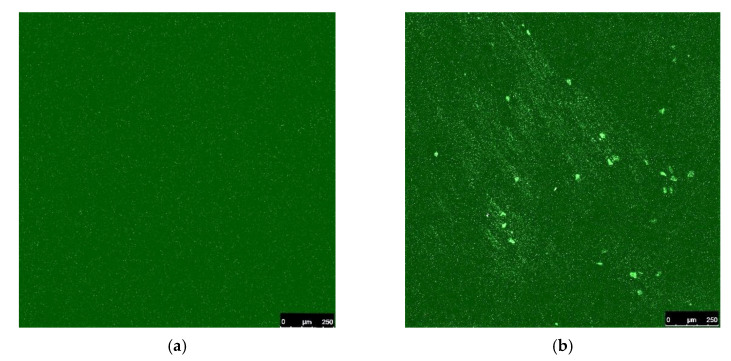
Rat brain slices: (**a**) after administration of free rhodamine B (2.5 mg/kg); (**b**) after intravenous injection of rhodamine B (2.5 mg/kg) in 20 mM IA-16/PC (0.029/1) liposomes.

To test the ability of imidazolium liposomes to penetrate through the BBB, using the IA-16/PC (surfactant/lipid molar ratio of 0.029/1) system as an example, the method of confocal fluorescence microscopy was used. Examination of rat brain cross-section samples was performed after intravenous administration of the liposomal form of rhodamine B. Free rhodamine B was used as a control (Figure 4a). The figure shows that rhodamine B in the free form does not pass through the BBB, as evidenced by the complete absence of luminescence in the brain region (Figure 4a). While liposomal rhodamine B enters the rat brain, as evidenced by the glow in the photographs (Figure 4b).

### 3.4. Preparation, Characteristics of 2-PAM-Loaded Liposomes: In Vitro Release of 2-PAM

Analysis of the zeta potential, particle size, and release rate of rhodamine B as well as the EE led to the selection of IA-16/PC for 2-PAM encapsulating. Furthermore, the amount of 2-PAM was optimized for encapsulation in liposomes (Table 3). It can be seen that the loading of 2-PAM into liposomes, regardless of its amount, leads to the formation of larger aggregates compared with empty systems (D_H_ is in the range 107–131 nm). The aggregates have a narrow size distribution (PdI does not exceed 0.11) and approximately the same EE (~70–74%). However, when loading different amounts of 2-PAM, such parameters as zeta potential and loading capacity (LC) are very different. LC is the amount of loaded drug substance per unit of weight of the nanoparticle, which indicates the percentage of the weight of the nanoparticle that is associated with the encapsulated drug. When 2-PAM is loaded into liposomes with a concentration of 20 mg/mL, this value is at the level of 91–96%, and when 2-PAM has a concentration of 10 mg/mL, the LC is 46–48% (Table 3).

**Figure 5:**
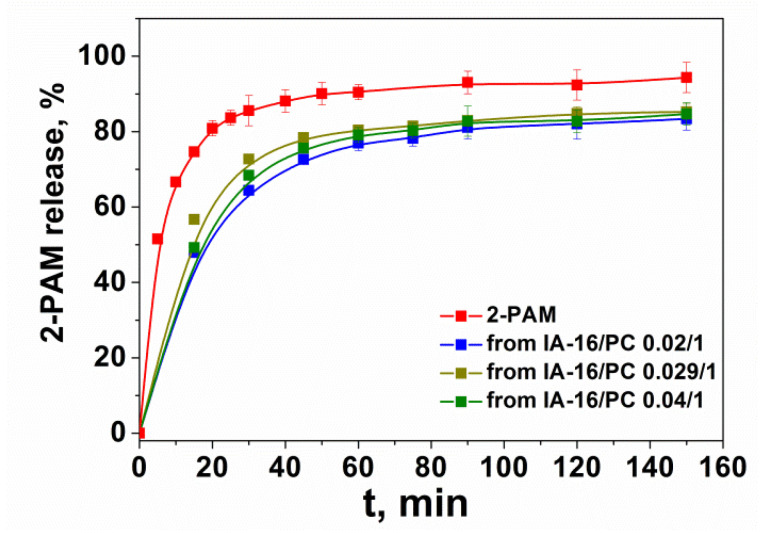
In vitro 2-PAM release from IA-16/PC modified liposomes using the dialysis bag method (*n* = 3); C (2-PAM) = 10 mg/mL, phosphate buffer (0.025 M), pH = 7.4, 37 °C.

The dialysis method was applied to study the release of 2-PAM in vitro (Figures S8 and S9). The release of 2-PAM from aqueous solution and from liposomes is presented in Figure 5 and Figure S10.

### 3.5. Analysis of Hemolytic Activity and Hemagglutination Caused by Modified Liposomes Loaded with 2-PAM

Hemolysis of erythrocytes using liposomal dosage forms is a process of great fundamental and practical importance. The hemolytic activity of the systems can also be used as a means for their toxicological assessment. In this regard, IA-16/PC liposomes (surfactant/lipid molar ratio of 0.029/1) loaded with 2-PAM (C = 10 mg/mL) were tested for their ability to induce hemolysis in erythrocytes and their concentration (HC_50_) that caused hemolysis in 50% of erythrocytes (Table 4). It was shown that the studied systems have insignificant hemolytic activity, which decreases with dilution. This allows for the use of these liposomes for in vivo experiments.

**Figure 6:**
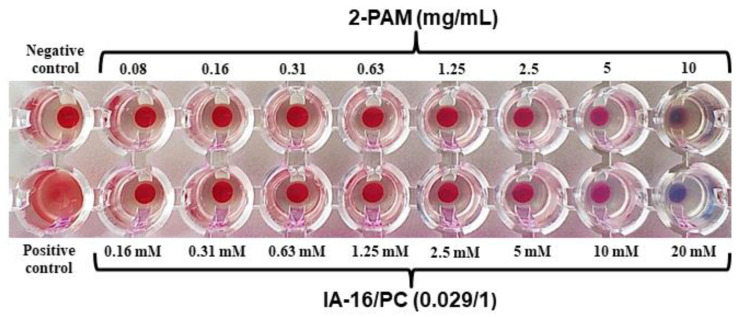
Photograph of microplate wells containing blood samples with addition of free 2-PAM and IA-16/PC formulation, C (2-PAM) = 10 mg/mL.

**Figure 7:**
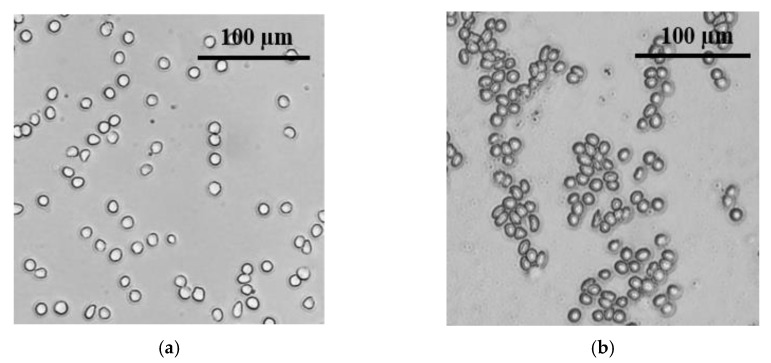
(**a**) Effect of cationic liposomes IA-16/PC (surfactant/lipid molar ratio of 0.029/1) on agglutination of erythrocytes was observed by fluorescent microscopy in phase contrast mode in liposome solutions with the highest lipid concentration; (**b**) in mixture of type A (II) and B (III) erythrocytes—positive agglutination control.

Also, IA-16/PC liposomal systems (surfactant/lipid molar ratio of 0.029/1) loaded with 2-PAM (C = 10 mg/mL) were tested for the ability to cause hemagglutination. Hemagglutination is the adhesion and precipitation of erythrocytes under the influence of bacteria, viruses, toxins, etc., that can be adsorbed on the surface of erythrocytes. A negative test shows a round red button, which corresponds to normal red blood cells in saline (Figure 6, Negative control). For agglutinating erythrocytes, a cloudy red suspension is observed (Figure 6, Positive control). It can be seen that the addition of even concentrated IA-16/PC liposomal solutions loaded with 2-PAM (C = 10 mg/mL) to erythrocytes does not cause coalescence and adhesion of particles. Also, using the IA-16/PC system as an example, the absence of particle adhesion was proven by the method of fluorescence microscopy in phase contrast mode (Figure 7).

### 3.6. Reactivation of Brain AChE In Vivo

**Figure 8:**
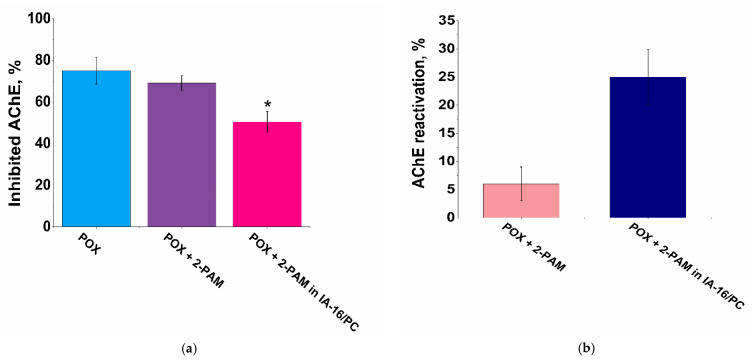
The study of brain AChE inhibition (**a**) and reactivation (**b**) in vivo. Mean AChE activity in brain homogenates was measured in a control group of rats (taken as 100%) after poisoning by POX and after injection of free 2-PAM (dose of 2-PAM was 25 mg/kg) and 2-PAM-loaded IA-16/PC liposomes (dose of 2-PAM was 25 mg/kg). Each point represents the mean percent of AChE inhibition in each experimental group. Data presented as mean ± standard error from 5 brain samples; * indicates significant difference by the Mann–Whitney test (*p* < 0.05) compared with the level of brain AChE inhibition detected 1 hour after POX injection.

The effectiveness of modified liposomes loaded with 2-PAM was evaluated to reactivate brain AChE after poisoning with sublethal dose of POX (0.6 mg/kg, intraperitoneally). It has been shown that free 2-PAM cannot reactivate brain AChE (Figure 8). However, administration of 2-PAM in liposomal form significantly reduces the inhibition of brain AChE from 75 ± 6% to 50 ± 5% in the case of the IA-16/PC (surfactant/lipid molar ratio of 0.029/1) (Figure 8a). Thus, liposomes IA-16/PC with 2-PAM reactivate brain AChE by 25 ± 5% (Figure 8b). High values of AChE reactivation in the brain are another preliminary evidence of BBB penetration that signify the potential of IA-16/PC liposomes to enhance current treatment of OP-induced neurodegeneration.

### 3.7. 2-PAM Pharmacokinetics in Rat Plasma and Brain Tissue

**Figure 9:**
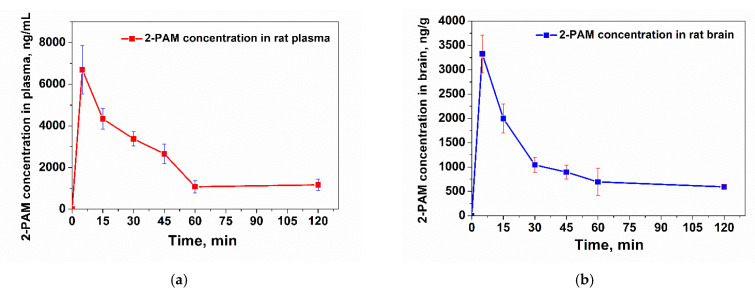
Concentration of 2-PAM in blood plasma (**a**) and brains (**b**) of rats after intravenous injection of 2-PAM in IA-16/PC liposomes (25 mg/kg), determined by HPLC-ESI-MS. Each point represents the mean ± standard deviation for five rats.

In the next stage, pharmacokinetic studies were carried out for IA-16/PC liposomal solutions (surfactant/lipid molar ratio of 0.029/1) loaded with 2-PAM. The concentrations of 2-PAM in the blood plasma and brains of rats were analyzed using HPLC-ESI-MS. The kinetic dependences of the mean concentration of 2-PAM in the plasma and brains of rats after intravenous administration of the liposomal form of 2-PAM (dose of 2-PAM: 25 mg/kg) are shown in Figure 9. It is shown that the maximum concentrations of 2-PAM after intravenous administration in blood plasma and brain tissue were reached after 5 min and amounted to 6700 ng/mL and 3325 ng/g, respectively (Figure 9). The concentration of 2-PAM gradually decreased over 120 min.

### 3.8. Effect of Liposomes Loaded with 2-PAM on POX-Induced Neurotoxicity

The ability of IA-16/PC liposomes loaded with 2-PAM to reduce POX-induced neurotoxicity was studied using a previously described mice model [65]. It was shown that administration of POX (2 mg/kg, s.c.) without any antidote therapy leads to the death of all mice within a few minutes (Table 5). However, it was shown that all animals survived in the groups of mice that received 2-PAM and atropine, or IA-16/PC liposomes loaded with 2-PAM and atropine. Thus, both free 2-PAM and liposomal 2-PAM prevented the deaths of POX-poisoned mice with the same efficiency. However, free 2-PAM poorly penetrates through the BBB. Therefore, in the next set of experiments, we compared ability of free 2-PAM and IA-16/PC liposomes loaded with 2-PAM to reduce neuronal death in the entorhinal cortex and hippocampus of mice that survived after POX poisoning.

**Figure 10:**
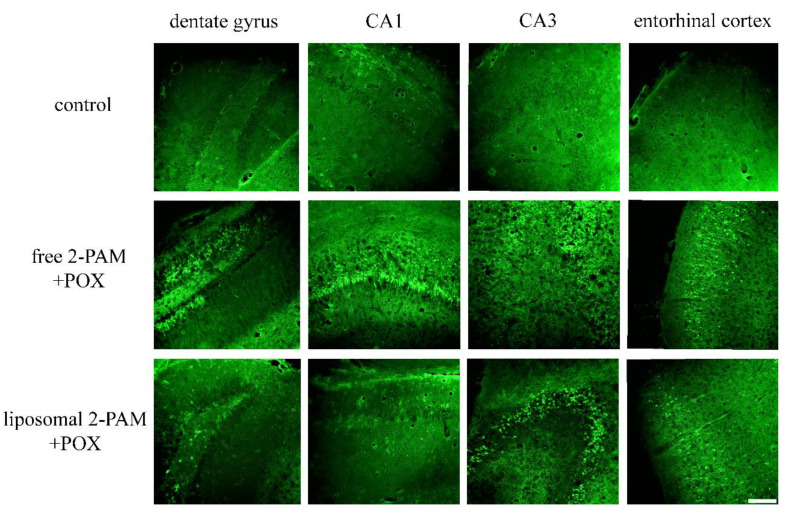
Representative microphotographs of Fluoro-Jade B staining showing the neuroprotective effect of liposomes loaded with 2-PAM following POX poisoning in the dentate gyrus, CA1, and CA3 areas of the hippocampus and in the entorhinal cortex of mice. Scale bar 100 μm.

Neuronal degeneration was examined using Fluoro-Jade B (FJB) staining. It was shown that brain sections of control mice exhibit only a few fluorescent FJB-labeled neurons (Table 6, Figure 10). Significant numbers of *diffuse* Fluoro-Jade B-positive cells, indicating apoptotic cells, were observed both in the entorhinal cortex and in all areas of the hippocampus of mice treated with free 2-PAM: the mean number of FJB-positive neurons per 1 mm^2^ significantly increased from 2.71 ± 0.27 to 12.46 ± 1.6 (*p* < 0.0001) in dentate gyrus, from 2.28 ± 0.29 to 17.27 ± 1.76 (*p* < 0.0001) in the CA1 zone of the hippocampus, from 1.89 ± 0.23 to 9.2 ± 0.91 (*p* < 0.0001) in the CA3 zone of the hippocampus, and from 1.78 ± 0.18 to 9.09 ± 0.57 (*p* < 0.0001) in the entorhinal cortex. Mice treated with liposomal 2-PAM showed significantly lower levels of FJB labeling in the dentate gyrus and CA1 region compared with the group of mice treated with free 2-PAM. In the dentate gyrus, the average number of FJB-stained neurons was 4.74 ± 0.46 per 1 mm^2^ (*p* < 0.015), and in the CA1 region of the hippocampus, it was 7.68 ± 0.91 per 1 mm^2^ (*p* < 0.014). 

## 4. Discussion

The BBB is a major barrier to effective drug delivery to the brain. The BBB consists of brain capillary endothelial cells and tight junctions between them, which prevent the penetration of almost all macromolecules and more than 98% of low-molecular-weight drugs [69,70]. Therefore, the development of targeted liposomes capable of crossing the BBB and delivering therapeutic molecules to the brain is of great interest [71]. Attempts to encapsulate OPs antidotes in nanoparticles have been made before, and the possibility of nanoscale drug delivery systems to act as carriers for AChE reactivators is discussed in detail, for example, in the reviews of [12,72]. For this purpose, different nanocarriers can be used, including liposomes [73], solid-lipid nanoparticles [19,20,74], human serum albumin nanoparticles [75], and mesoporous silica nanoparticles [76]. Herein, we continued our work in this direction to increase the efficiency of AChE reactivation upon the modification of liposomes with other surfactants and to study the mechanism of the neuroprotective action of the formulated 2-PAM. The size and charge of particles play a key role in their clearance, circulation time in the blood, and ability to pass through the BBB. In particular, it has been shown that nanocontainers smaller than 200 nm have increased circulation in the blood and, therefore, are in contact with the BBB for a longer time when the drug is absorbed by the brain [77]. In our case, all obtained liposomes were about 100–110 nm in size, which may allow them to circulate in the blood for a long time and be better absorbed by BBB endothelial cells. The effect of the surface charge of liposomes on the ability of particles to be absorbed by the brain tissues was investigated by injecting cationic, anionic, and neutral liposomal systems into the carotid arteries of rats [78]. It was shown that charged liposomes interacted more effectively with cells and that cationic liposomes had the highest concentration in the brain [78,79]. The obtained liposomes had high zeta potential; therefore, they should have a high ability to be absorbed by brain cells. 

In the case of systems loaded with rhodamine B, the formation of liposomes with optimal physicochemical parameters (size, zeta potential) was also observed. Analysis of the rate of rhodamine B release from modified liposomes showed that an interesting trend is observed for IA-n/PC systems. When liposomes are modified with lower homologues (IA-10, IA-12), the release rate of the probe strongly depends on the molar ratio of the surfactant/lipid. The more surfactants in the system, the faster the release occurs. With an increase in the length of the hydrocarbon tail of amphiphiles, the difference in the rate of release of the substrate, depending on the molar ratio of the components, decreases. Furthermore, for systems based on IA-16/PC and IA-18/PC, the rate of rhodamine B release does not depend on the molar ratio of the components. This phenomenon may be due to the fact that the lower homologues of imidazolium surfactants tend to loosen the lipid bilayer, while the higher homologues, on the contrary, stabilize the lipid bilayer [47,49]. 

As mentioned earlier, the BBB prevents the penetration of various substances into the central nervous system. It is believed that cationic liposomes can penetrate through the BBB due to the mechanism of adsorption-mediated transcytosis. This mechanism can be triggered by electrostatic interaction between positively charged components of a liposome and negatively charged microregions of the endothelial cells of the BBB capillaries [33,39,40,80]. Consequently, liposomes modified with an imidazolium surfactant can be used to load 2-PAM, the main limitation of which is insignificant passage through the BBB. Alternative antidote molecules that are able to cross the BBB are being continuously developed and tested on OPs-poisoned in vivo models [81]. In general, these are simpler alternatives to liposomal forms that may be difficult to prepare and develop, but many novel BBB-permeating acetylcholinesterase reactivators show signs of toxicity and non-specificity [72]. Liposomes may be used with these new drugs to even further improve their therapeutic efficacy. 

Further research focused on obtaining liposomal formulations loaded with the cholinesterase reactivator 2-PAM. 2-PAM is considered the gold standard in oxime therapy and is used in the treatment of acute poisoning caused by OPs [82]. However, 2-PAM shows limited treatment efficacy due to its low ability to cross the BBB and its rapid elimination from the body [83]. Therefore, it was chosen as a model substance for loading into liposomal nanocontainers. The values of LC for liposomes with 20 mg/mL 2-PAM are twice those for the system with 10 mg/mL 2-PAM. This makes systems with 2-PAM loading of 20 mg/mL more promising in terms of the amount of substrate in liposomes. However, 2-PAM is capable of lowering the zeta potential of systems, and in the case of encapsulation in lipid formulations, a concentration of 2-PAM of 10 mg/mL lowers the zeta potential of particles by no more than 10 units, while a concentration of 20 mg/mL leads to reduction of the zeta potential of particles by up to 30 units. This can significantly affect the stability of the particles and their ability to pass through the BBB. Therefore, preference was given to particles with a 2-PAM loading of 10 mg/mL. 

Controlled and sustained drug release can be achieved by modifying the liposomal carriers, for example with polymers or surfactants [52,84]. In addition, compaction and loosening of the bilayer will act as competing processes. It was shown that unencapsulated 2-PAM is released from the dialysis bag within 1 h (about 92%). The rapid release of 2-PAM during dialysis in a phosphate buffer has been observed previously for liposomal systems [17,73] and may be due to the small size of this molecule. At the same time, its encapsulation into modified liposomes makes it possible to prolong the release time of the substrate to up to 3 h. 

Intravenously administered nanocontainers are in direct contact with the circulatory system for a long time and can cause unintended hemostatic and inflammatory reactions. Usually, positively charged nanocontainers can interact with the negatively charged erythrocyte plasma membrane, which subsequently leads to hemolysis [85]. The developed nanocontainers at high concentrations (10 mM) showed hemolysis of 20%, which indicates release of hemoglobin. The slight formulation-induced hemolysis may be due to the presence of cationic surfactants, which were used to formulate the vesicles. Thus, the low percentage of hemolysis indicates that the resulting systems were biocompatible and non-toxic for human blood and were suitable for intravenous delivery of 2-PAM.

Hemagglutination is the adhesion and precipitation of erythrocytes under the influence of bacteria, viruses, toxins, etc., that can be adsorbed on the surface of erythrocytes. The cytoplasmic membrane of erythrocytes consists of substances of a protein nature, i.e., agglutinogens, and proteins in the blood plasma are agglutinins. Despite their low content of cationic surfactant (surfactant/lipid molar ratio of 0.029/1), cationic liposomes have a fairly high zeta potential. However, in Figure 6, no positive control of agglutination is observed in any well. It is probable that cationic liposomes are more likely to interact with proteins in the blood plasma and not with proteins in the erythrocyte membrane. However, it is worth noting that this requires further research. Based on the studies of other authors, we can make the assumption that the proteins that are in the blood plasma reduce the high positive charge of cationic liposomes, which in turn prevents the aggregation of red blood cells [86].

**Scheme 1:**
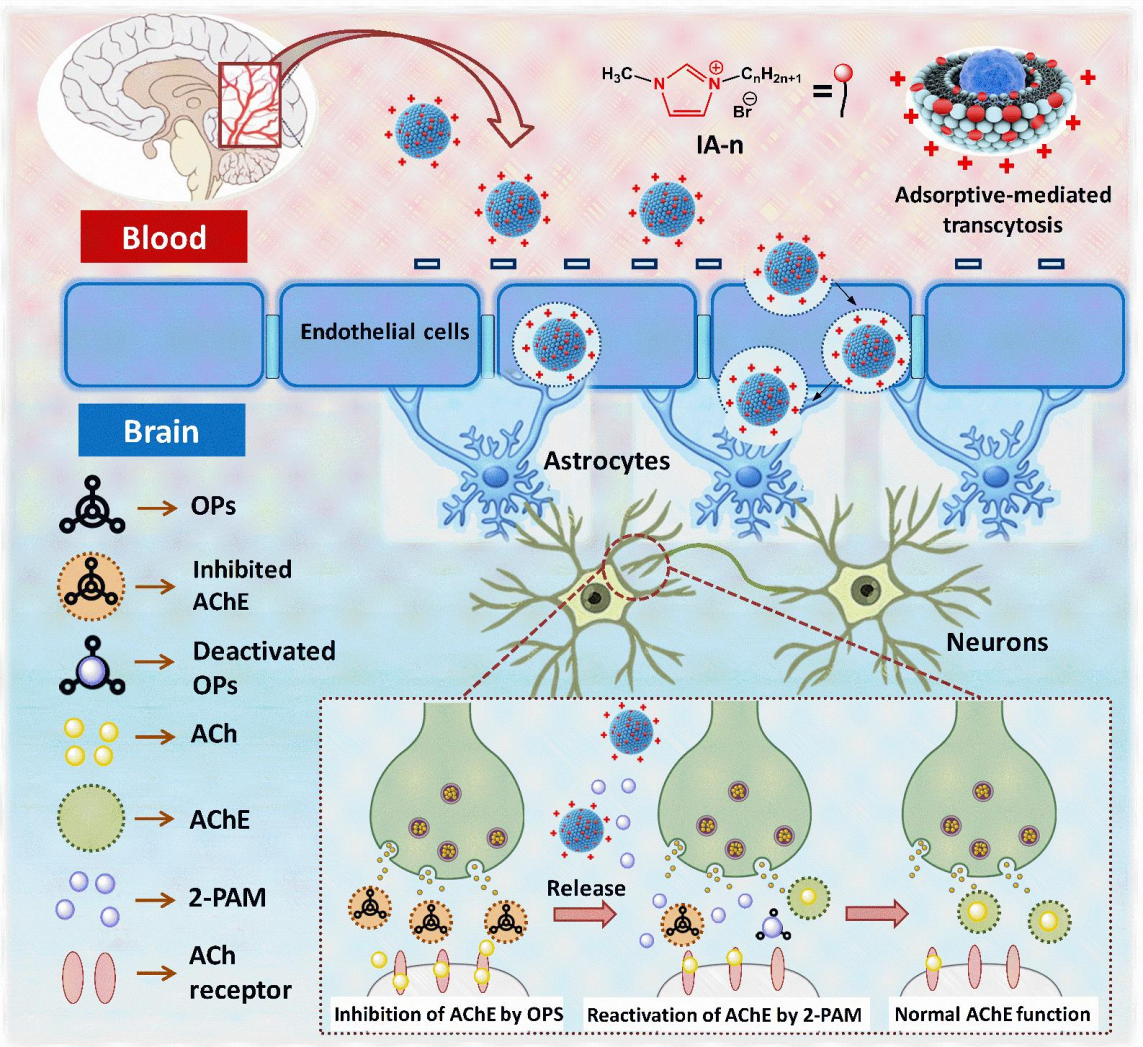
Mechanism of IA-16/PC liposome penetration of the blood–brain barrier and reactivation of the inhibited AChE.

The BBB consists of endothelial cells whose surface is mostly negatively charged. The low efficacy of free 2-PAM when administered intravenously is due to the fact that 2-PAM cannot penetrate through the BBB sufficiently to reach the CNS at concentrations required to reactivate AChE [17,73,87]. To overcome this limitation, research is being carried out in two directions: the synthesis of uncharged oximes and the encapsulation of existing oximes in nanocontainers capable of crossing the BBB. Modified cationic liposomes loaded with 2-PAM can penetrate into nerve tissues due to the positive charge of the liposomes and the similar structure of the lipid layer to the cell membrane. The presumed mechanism of action of 2-PAM-loaded IA-16/PC liposomes on OPs-inhibited AChE is shown in Scheme 1.

It should be noted that in the case of the liposomal form of 2-PAM, not all of the loaded oxime is associated with liposomes; part of it remains unencapsulated (EE for IA-16/PC is 72%, Table 3). Thus, the actual dose of liposomal 2-PAM in those cases was 18 mg/kg. There are few examples of 2-PAM delivery in liposomes. Cationic liposomes based on L-α-phosphatidylcholine and dihexadecylmethylhydroxyethylammonium bromide were used for intranasal administration of 2-PAM at a dose of 7 mg/kg [73]. 2-PAM-loaded liposomes reactivated 12% of brain AChE. Liposomes modified with hydroxyethyl bearing gemini surfactants were used for the intravenous administration of 2-PAM at a dose of 25 mg/kg [17]. Liposomal 2-PAM was found to reactivate 27% of brain AChE. Therefore, the new cationic IA-16/PC liposomes are optimal in terms of the administered dose and the achieved effect of AChE reactivation equal to 25%.

To evaluate the ability of 2-PAM-loaded cationic IA-16/PC liposomes to target and cross the BBB, pharmacokinetic studies were performed in brain tissues and plasma. Comparison of the pharmacokinetic dependences of free [17] and liposomal 2-PAM (in IA-16/PC liposomes, the ratio is 0.029/1) (Figure 9b) in rat brains suggests that encapsulated oxime penetrates through the BBB much more efficiently compared to the free form. Higher 2-PAM concentration in the case of the liposomal form is observed over the whole period that was monitored by HPLC. It should be noted that previously, after free 2-PAM (25 mg/kg) injection, the maximum concentration in the brain tissue was also measured at 5 min after injection and amounted to ~1100 ng/g [17]; consequently, at 60 min after injection, 2-PAM presence was not detectable. In this work, a threefold increase in the concentration of liposomal 2-PAM in the brain (3325 ng/g) compared with free 2-PAM is observed. It is also notable that the influence of the vesicle composition on the passage of the antidote into the brain. It was shown that 2-PAM in imidazolium liposomes penetrates into the brain 30% more (3325 ng/g) effectively than 2-PAM in liposomes modified by a gemini surfactant (2250 ng/g), which suggests a decisive role for the structure of the head group of the cationic surfactant. Also, modification of liposomes with imidazolium surfactants prolongs the retention time of 2-PAM in the brain by up to 2 h compared with gemini surfactants [17]. Most likely, the monocationic imidazolium surfactant promotes the formation of more densely packed vesicles, which allows substrate retention for a longer time.

Plots of 2-PAM concentration versus time can be analyzed using the two-compartment pharmacokinetic model equation: ${\left[C\right]}_{t}=Ae^{-\alpha t}+Be^{-\beta t}$, where [*C*]*_t_* is the 2-PAM concentration in plasma at time *t*, and *α* and *β* are the rate constants of distribution and excretion, respectively [19]. However, in our case, the *α* distribution phase is very fast, so we used the first-order kinetic equation, which takes into account only the elimination phase. The pharmacokinetic parameters of 2-PAM were calculated and presented in Table 7. The half-life of 2-PAM from blood plasma was about 23 min, and it was about 52 min for 2-PAM from the brain. It is worth noting that for free 2-PAM, the blood half-life was only 10.7 min. It should also be noted that in the case of liposomes modified with gemini surfactants, the half-life of 2-PAM in plasma was 12.7 min [17], and in solid lipid particles, it was 80 min [74]. Thus, the structure of cationic surfactants and the charges of particles play a significant role in the creation of nanocontainers targeted toward the BBB.

The half-life can be correlated with hemocompatibility to assess the effect of particles on the blood. First, in the analysis of hemolytic activity and hemagglutination, a suspension of erythrocytes was incubated with particles for 1 h, and the half-life from plasma is 23 min, while the half-life from the brain is 52 min. Therefore, the particles can be expected to circulate freely in the blood without clumping and without causing hemolysis. On the other hand, the study of hemagglutination was carried out at a concentration of 20 mM and subsequent dilution. Furthermore, the same concentration of liposomes was administered in pharmacokinetic experiments. In Figure 6, no positive agglutination control is observed in any well (even at the highest concentration). Therefore, this may also indicate the absence of the influence of particles on the blood and their free circulation.

Neuroprotection is an important aspect of OPs poisoning because it is the treatment of long-term neuronal damage induced by phosphates. Patel et al. investigated an antioxidant porphyrin derivative that was able to significantly lower neurodegeneration in a diisopropyl fluorophosphate poisoning model due to its antioxidant abilities [88]. In our case, however, no deliberate antioxidant treatment is performed, hence the neuroprotective effect may be explained by increased 2-PAM presence in the brain, which prevents long-term AChE inhibition. Non-liposomal 2-PAM was used in that work but its efficacy was impaired due to the difficulty of BBB passage. Similarly, free 2-PAM treatment was not able to protect neurons from POX-induced damage in work by Chambers et al. [81]. Thus, intravenous administration of liposomes loaded with 2-PAM can significantly reduce POX-induced neuronal death in the CA1 region and dentate gyrus of the mouse hippocampus.

## 5. Conclusions

For the first time, cationic liposomes modified with imidazolium surfactants were obtained for the treatment of OPs poisoning in animal models. High stability, high values of encapsulation efficiency and loading capacity, low hemolytic activity, and low hemagglutination induction were shown for all systems. Imidazolium liposomes were able to penetrate through the BBB and deliver 2-PAM into the brain. The use of modified liposomes makes it possible to achieve a high level of AChE reactivation (25 ± 5%). The half-life of 2-PAM in blood plasma after intravenous administration in the liposomal form was about 24 min, and it was about 52 min in the brain, while the half-life of free 2-PAM in blood plasma was only 10.7 min. For the first time, an assessment of the ability of imidazolium liposomes loaded with 2-PAM to reduce the death of neurons in the brain of mice caused by OPs poisoning was carried out. It was found that in the CA1 zone of the hippocampus, there was a decrease of damaged Fluoro-Jade-positive cells from 17.27 ± 1.76 to 7.68 ± 0.91 cells per mm^2^, and in the dentate gyrus of the hippocampus from 12.46 ± 1.6 to 4.75 ± 0.46 cells per mm^2^. Following the successful application of 2-PAM for the treatment of OPs poisoning, it is of interest to develop liposomal forms of other oximes to improve their efficacy. Expansion of the variety of oximes will contribute to the breadth of treatment tools for poisoning with various types of OPs, not only paraoxon.

## Figures and Tables

**Table 1:** Physicochemical characteristics of liposomes based on PC modified by IA-n at various surfactant/lipid molar ratios, hydrocarbon tail length, and storage times: hydrodynamic diameter (D_H_, nm), polydispersity index (PdI), and zeta potential (ζ, mV), 25 °C.

System	Surfactant/PC Molar Ratio	1 Day			3 Months		
		D_H_, nm	PdI	ζ, mV	D_H_, nm	PdI	ζ, mV
PC	-	105 ± 2	0.205 ± 0.013	−5 ± 1	not stable		
IA-10/PC	0.02/1	107 ± 1	0.077 ± 0.008	22 ± 1	118 ± 5	0.231 ± 0.024	17 ± 1
IA-10/PC	0.029/1	110 ± 1	0.068 ± 0.010	27 ± 2	121 ± 4	0.141 ± 0.010	25 ± 2
IA-10/PC	0.04/1	107 ± 1	0.116 ± 0.010	31 ± 1	110 ± 2	0.099 ± 0.012	22 ± 1
IA-12/PC	0.02/1	111 ± 1	0.123 ± 0.012	27 ± 1	118 ± 1	0.168 ± 0.015	24 ± 1
IA-12/PC	0.029/1	109 ± 2	0.106 ± 0.010	30 ± 1	115 ± 3	0.105 ± 0.011	42 ± 1
IA-12/PC	0.04/1	107 ± 2	0.178 ± 0.015	43 ± 2	108 ± 1	0.184 ± 0.009	44 ± 1
IA-14/PC	0.02/1	116 ± 1	0.158 ± 0.012	45 ± 1	109 ± 1	0.083 ± 0.006	38 ± 2
IA-14/PC	0.029/1	109 ± 1	0.129 ± 0.011	52 ± 2	121 ± 1	0.054 ± 0.005	49 ± 2
IA-14/PC	0.04/1	109 ± 1	0.164 ± 0.010	56 ± 1	103 ± 1	0.081 ± 0.013	41 ± 3
IA-16/PC	0.02/1	106 ± 1	0.136 ± 0.012	41 ± 2	107 ± 1	0.095 ± 0.016	35 ± 2
IA-16/PC	0.029/1	110 ± 2	0.134 ± 0.015	51 ± 2	107 ± 1	0.108 ± 0.020	56 ± 2
IA-16/PC	0.04/1	112 ± 2	0.164 ± 0.010	59 ± 1	106 ± 1	0.123 ± 0.013	64 ± 3
IA-18/PC	0.02/1	110 ± 1	0.074 ± 0.010	43 ± 1	108 ± 1	0.057 ± 0.024	39 ± 1
IA-18/PC	0.029/1	103 ± 1	0.068 ± 0.008	54 ± 2	102 ± 1	0.094 ± 0.010	56 ± 2
IA-18/PC	0.04/1	107 ± 2	0.124 ± 0.010	57 ± 1	106 ± 1	0.077 ± 0.012	45 ± 1

**Table 2:** Physicochemical characteristics of liposomes based on PC modified by IA-n loaded with rhodamine B at various surfactant/lipid molar ratios, IA-n hydrocarbon tail lengths, and storage times: encapsulation efficiency (EE, %), hydrodynamic diameter (D_H_, nm), polydispersity index (PdI), and zeta potential (ζ, mV), 25 °C.

Rhodamine B								
System	Surfactant/ PC Molar Ratio	EE,%	1 Day			2 Months		
			D_H_, nm	PdI	ζ, mV	D_H_, nm	PdI	ζ, mV
PC	-	34 ± 1	105 ± 2	0.055 ± 0.002	−3 ± 1	not stable		
IA-10/PC	0.02/1	72 ± 1	107 ± 3	0.101 ± 0.026	45 ± 1	111 ± 2	0.075 ± 0.012	31 ± 4
IA-10/PC	0.029/1	72 ± 2	102 ± 2	0.049 ± 0.006	50 ± 1	129 ± 3	0.176 ± 0.014	29 ± 2
IA-10/PC	0.04/1	70 ± 1	101 ± 1	0.073 ± 0.044	60 ± 1	93 ± 3	0.218 ± 0.006	24 ± 1
IA-12/PC	0.02/1	70 ± 1	102 ± 2	0.080 ± 0.017	32 ± 1	116 ± 2	0.183 ± 0.021	23 ± 1
IA-12/PC	0.029/1	69 ± 2	108 ± 2	0.137 ± 0.006	37 ± 1	113 ± 3	0.190 ± 0.033	24 ± 1
IA-12/PC	0.04/1	69 ± 1	101 ± 2	0.088 ± 0.025	48 ± 1	119 ± 1	0.194 ± 0.013	27 ± 1
IA-14/PC	0.02/1	67 ± 3	103 ± 1	0.108 ± 0.008	48 ± 1	112 ± 3	0.089 ± 0.001	23 ± 1
IA-14/PC	0.029/1	69 ± 1	101 ± 2	0.068 ± 0.016	54 ± 1	105 ± 2	0.197 ± 0.034	26 ± 1
IA-14/PC	0.04/1	69 ± 2	102 ± 1	0.082 ± 0.011	57 ± 1	105 ± 1	0.161 ± 0.030	31 ± 3
IA-16/PC	0.02/1	80 ± 1	112 ± 2	0.107 ± 0.039	43 ± 1	121 ± 3	0.211 ± 0.011	21 ± 1
IA-16/PC	0.029/1	81 ± 1	109 ± 1	0.093 ± 0.013	53 ± 1	141 ± 2	0.194 ± 0.100	25 ± 1
IA-16/PC	0.04/1	78 ± 2	104 ± 2	0.179 ± 0.038	65 ± 1	136 ± 1	0.191 ± 0.030	28 ± 3
IA-18/PC	0.02/1	76 ± 1	112 ± 2	0.151 ± 0.007	39 ± 3	138 ± 3	0.193 ± 0.011	22 ± 1
IA-18/PC	0.029/1	77 ± 3	106 ± 2	0.090 ± 0.012	52 ± 1	128 ± 3	0.211 ± 0.011	26 ± 1
IA-18/PC	0.04/1	76 ± 1	105 ± 2	0.086 ± 0.009	59 ± 1	120 ± 3	0.172 ± 0.033	48 ± 1

**Table 3:** Physicochemical characteristics of liposomes based on PC modified by IA-16 loaded with 2-PAM at various surfactant/lipid molar ratios and different loadings of the substrate: encapsulation efficiency (EE, %), loading capacity (LC, %); hydrodynamic diameter (D_H_, nm), polydispersity index (PdI), and zeta potential (ζ, mV), 25 °C.

2-PAM							
System	Surfactant/PC Molar Ratio	Initial Concentration of Substrate, mg/mL	EE, %	LC, %	D_H_, nm	PdI	ζ, mV
PC	-		34 ± 1		105 ± 2	0.055 ± 0.002	−3 ± 1
IA-16/PC	0.02/1	10	74 ± 1	48 ± 1	122 ± 1	0.110 ± 0.018	33 ± 1
IA-16/PC	0.029/1	10	72 ± 1	46 ± 1	109 ± 2	0.088 ± 0.030	42 ± 1
IA-16/PC	0.04/1	10	72 ± 1	46 ± 1	112 ± 2	0.082 ± 0.008	49 ± 1
IA-16/PC	0.02/1	20	73 ± 1	96 ± 1	131 ± 2	0.088 ± 0.024	17 ± 1
IA-16/PC	0.029/1	20	73 ± 1	96 ± 1	127 ± 1	0.075 ± 0.016	23 ± 1
IA-16/PC	0.04/1	20	74 ± 1	97 ± 1	131 ± 1	0.080 ± 0.025	28 ± 1

**Table 4:** Evaluation of the hemolytic activity of IA-16/PC liposomal system (surfactant/lipid molar ratio of 0.029/1) loaded with 2-PAM (C = 10 mg/mL).

System	Concentration, mM	Hemolysis, %	HC_50_, mM
IA-16/PC + 2-PAM	10	20.9	>10
	5	12.0	
	2.5	5.1	
	1.25	2.1	
	0.625	0.9	
	0.313	0	
	0.156	0	

**Table 5:** Protection of mice against POX toxicity.

Group	*n*/N *
POX (2 mg/kg, s.c.)	0/12
Free 2-PAM (25 mg/kg, i.v.) + POX (2 mg/kg, s.c.)	12/12
Liposomal 2-PAM (25 mg/kg, i.v.) + POX (2 mg/kg, s.c.)	12/12

* *n*—number of rats survival after challenge; N—total number of rats in the group.

**Table 6:** Quantitative analyses of Fluoro-Jade B labeling in the dentate gyrus, the CA1 and CA3 zones of the hippocampus, and in the entorhinal cortex of mice poisoned by POX and treated with free 2-PAM or liposomal 2-PAM.

Group	Fluoro-Jade B Positive Cells/mm^2^			
	Dentate Gyrus	CA1	CA3	Entorhinal Cortex
Control	2.71 ± 0.27	2.28 ± 0.29	1.89 ± 0.23	1.78 ± 0.18
Free 2-PAM (25 mg/kg, i.v.) + POX (2 mg/kg, s.c.)	12.46 ± 1.6 ***	17.27 ± 1.76 ***	9.77 ± 1.17 ***	10.48 ± 0.69 ***
Liposomal 2-PAM (25 mg/kg, i.v.) + POX (2 mg/kg, s.c.)	4.75 ± 0.46 **^#^	7.68 ± 0.91 ***^#^	9.2 ± 0.91 ***	9.09 ± 0.57 ***

Data represent the mean value ± SEM from 48 brain sections in each group. ** *p* < 0.01, *** *p* < 0.001 compared with the control group; # *p* < 0.05 compared with the group treated with free 2-PAM. Statistical analysis was performed using the Mann-Whitney test.

**Table 7:** Pharmacokinetic parameters obtained by analysis of rat tissues after intravenous injection of 2-PAM in IA-16/PC liposomes (dose 25 mg/kg). Results presented as mean ± standard error for five rats.

	β (min^−1^)	t_1/2β_ (min)
Plasma	0.0296 ± 0.002	23.4 ± 0.02
Brain	0.0134 ± 0.001	51.7 ± 0.01
t_1/2β_ = ln2/β, t_1/2β_—elimination half-life		

## Data Availability

Not applicable.

## Supplementary Materials

The following supporting information can be downloaded at: https://www.mdpi.com/article/10.3390/pharmaceutics14091950/s1, Figure S1: Calibration curve of 2-PAM in rat plasma (a) and in rat brain (b); Figure S2. The absorption spectra of rhodamine B at different release time intervals for modified IA-10/PC liposomes, molar ratio of components: 0.02/1; 0.029/1; 0.04/1; 37 °C, phosphate buffer (0.025 M), pH = 7.4; Figure S3: The absorption spectra of rhodamine B at different release time intervals for modified IA-12/PC liposomes, molar ratio of components: 0.02/1; 0.029/1; 0.04/1; 37 °C, phosphate buffer (0.025 M), pH = 7.4; Figure S4: The absorption spectra of rhodamine B at different release time intervals for modified IA-14/PC liposomes, molar ratio of components: 0.02/1; 0.029/1; 0.04/1; 37 °C, phosphate buffer (0.025 M), pH = 7.4; Figure S5: The absorption spectra of rhodamine B at different release time intervals for modified IA-16/PC liposomes, molar ratio of components: 0.02/1; 0.029/1; 0.04/1; 37 °C, phosphate buffer (0.025 M), pH = 7.4; Figure S6: The absorption spectra of rhodamine B at different release time intervals for modified IA-18/PC liposomes, molar ratio of components: 0.02/1; 0.029/1; 0.04/1; 37 °C, phosphate buffer (0.025 M), pH = 7.4; Figure S7: In vitro rhodamine B release from mixed liposomes at various surfactant/lipid molar ratios: IA-12/PC; IA-14/PC; IA-18/PC; phosphate buffer (0.025 M), pH 7.4, 37 °C; Figure S8: The absorption spectra of 2-PAM at different release time intervals for modified IA-16/PC liposomes, molar ratio of components: 0.02/1; 0.029/1; 0.04/1; 37 °C, C (2-PAM) = 10 mg/mL; phosphate buffer (0.025 M), pH = 7.4; Figure S9: The absorption spectra of 2-PAM at different release time intervals for modified IA-16/PC liposomes, molar ratio of components: 0.02/1; 0.029/1; 0.04/1; 37 °C, C (2-PAM) = 20 mg/mL; phosphate buffer (0.025 M), pH = 7.4; Figure S10: In vitro 2-PAM release from IA-16/PC-modified liposomes using the dialysis bag method (*n* = 3); C (2-PAM) = 20 mg/mL, phosphate buffer (0.025 M), pH 7.4, 37 °C.
