# Supplementary material for: Oxime Therapy for Brain AChE Reactivation and Neuroprotection after Organophosphate Poisoning
Source: Pharmaceutics. 2022 Sep 15;14(9):1950. doi: 10.3390/pharmaceutics14091950 (PMC9506492; doi:10.3390/pharmaceutics14091950)
Supplement: Supplementary file 1 [file pharmaceutics-14-01950-s001.zip › pharmaceutics-1865367-supplementary.pdf]

pharmaceutics

MDPI

Supplementary Materials: Oxime Therapy for Brain AChE Reactivation and Neuroprotection after Organophosphate Poisoning

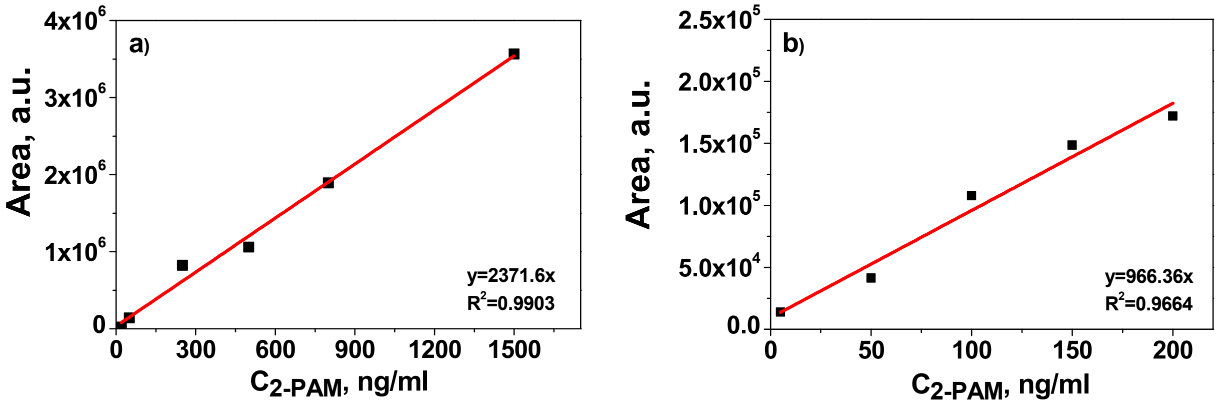

Figure S1. Calibration curve of 2-PAM in rat plasma (a) and in rat brain (b).

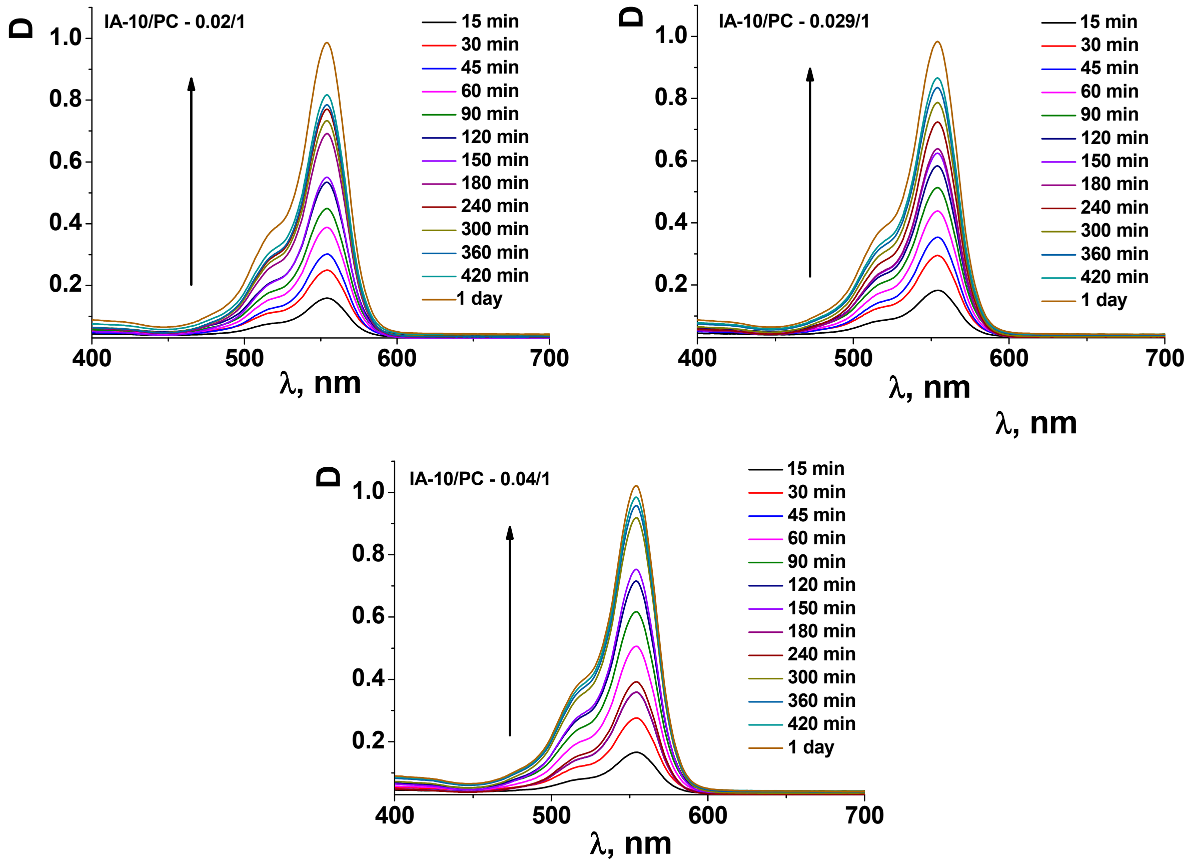

Figure S2. The absorption spectra of Rhodamine B at different release time intervals for modified IA-10/PC liposomes, molar ratio of components: 0.02/1; 0.029/1; 0.04/1; 37°C, phosphate buffer (0.025 M), pH = 7.4; the arrow indicates the direction of dialysis duration increasing.

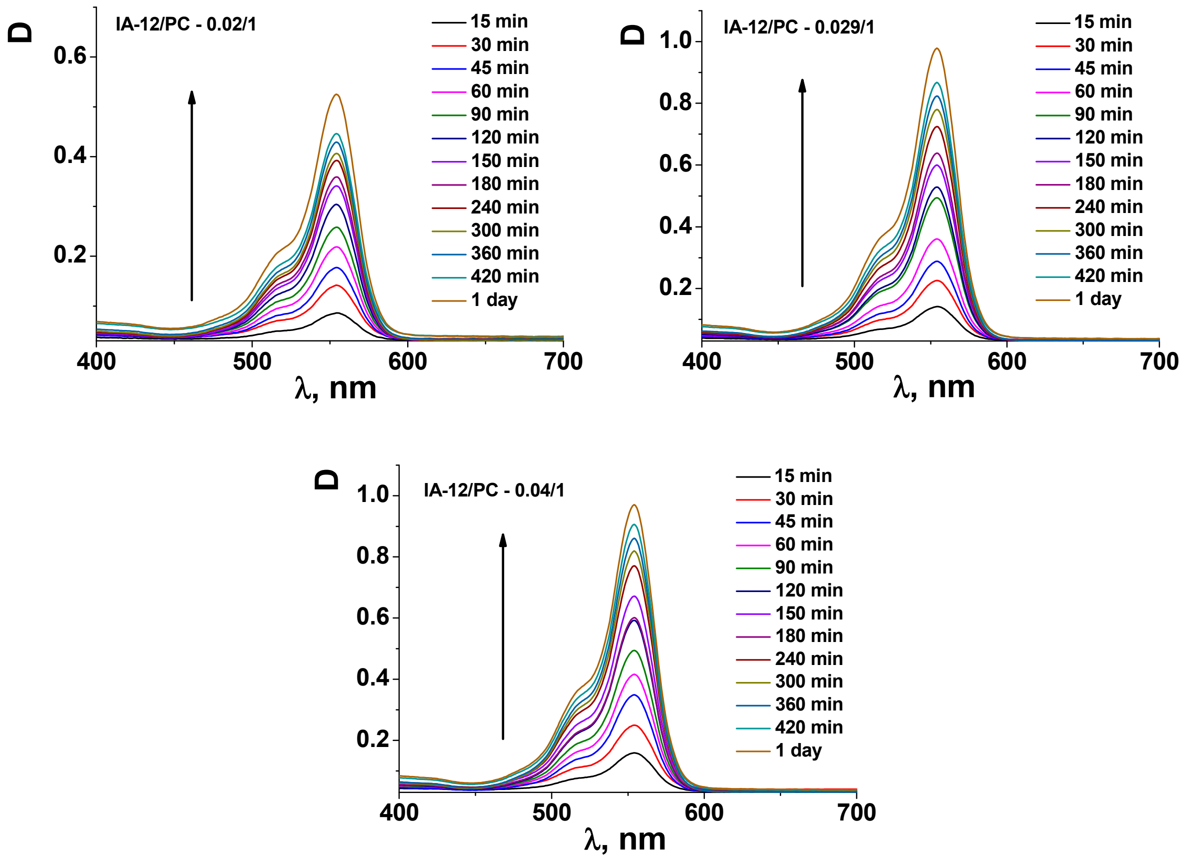

Figure S3. The absorption spectra of Rhodamine B at different release time intervals for modified IA-12/PC liposomes, molar ratio of components: 0.02/1; 0.029/1; 0.04/1; 37°C, phosphate buffer (0.025 M), pH = 7.4; the arrow indicates the direction of dialysis duration increasing.

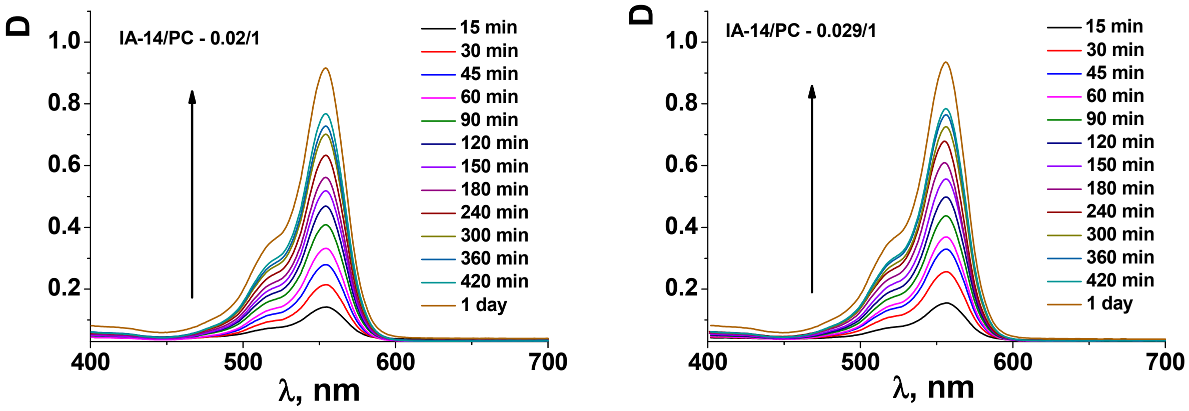

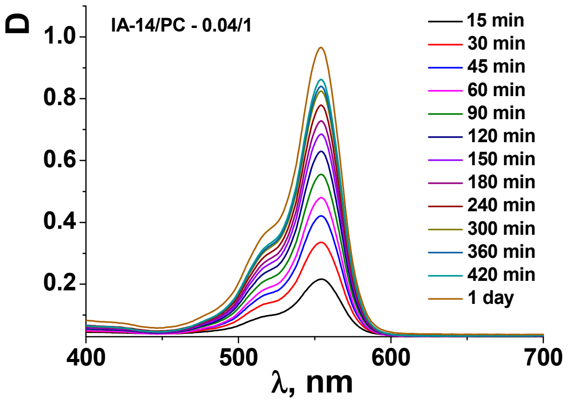

Figure S4. The absorption spectra of Rhodamine B at different release time intervals for modified IA-14/PC liposomes, molar ratio of components: 0.02/1; 0.029/1; 0.04/1; 37°C, phosphate buffer (0.025 M), pH = 7.4; the arrow indicates the direction of dialysis duration increasing.

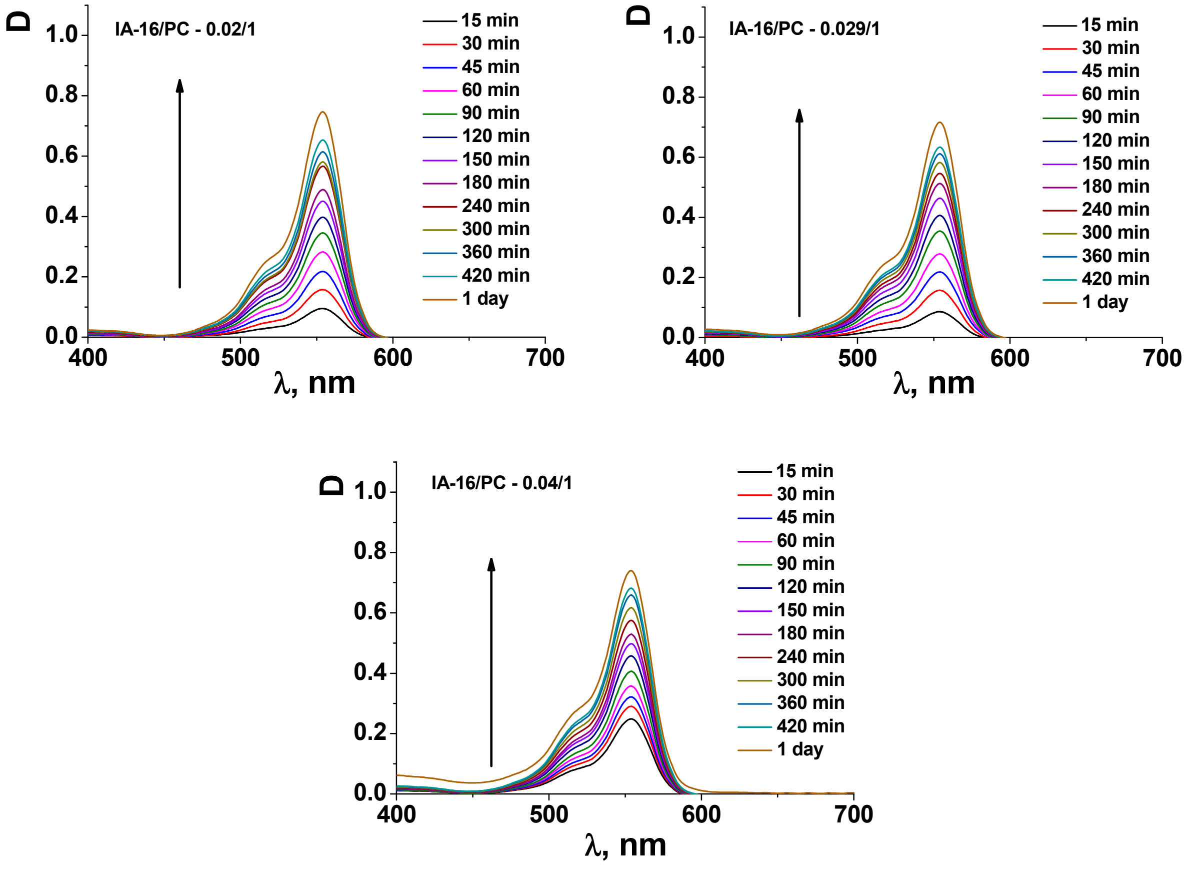

Figure S5. The absorption spectra of Rhodamine B at different release time intervals for modified IA-16/PC liposomes, molar ratio of components: 0.02/1; 0.029/1; 0.04/1; 37°C, phosphate buffer (0.025 M), pH = 7.4; the arrow indicates the direction of dialysis duration increasing.

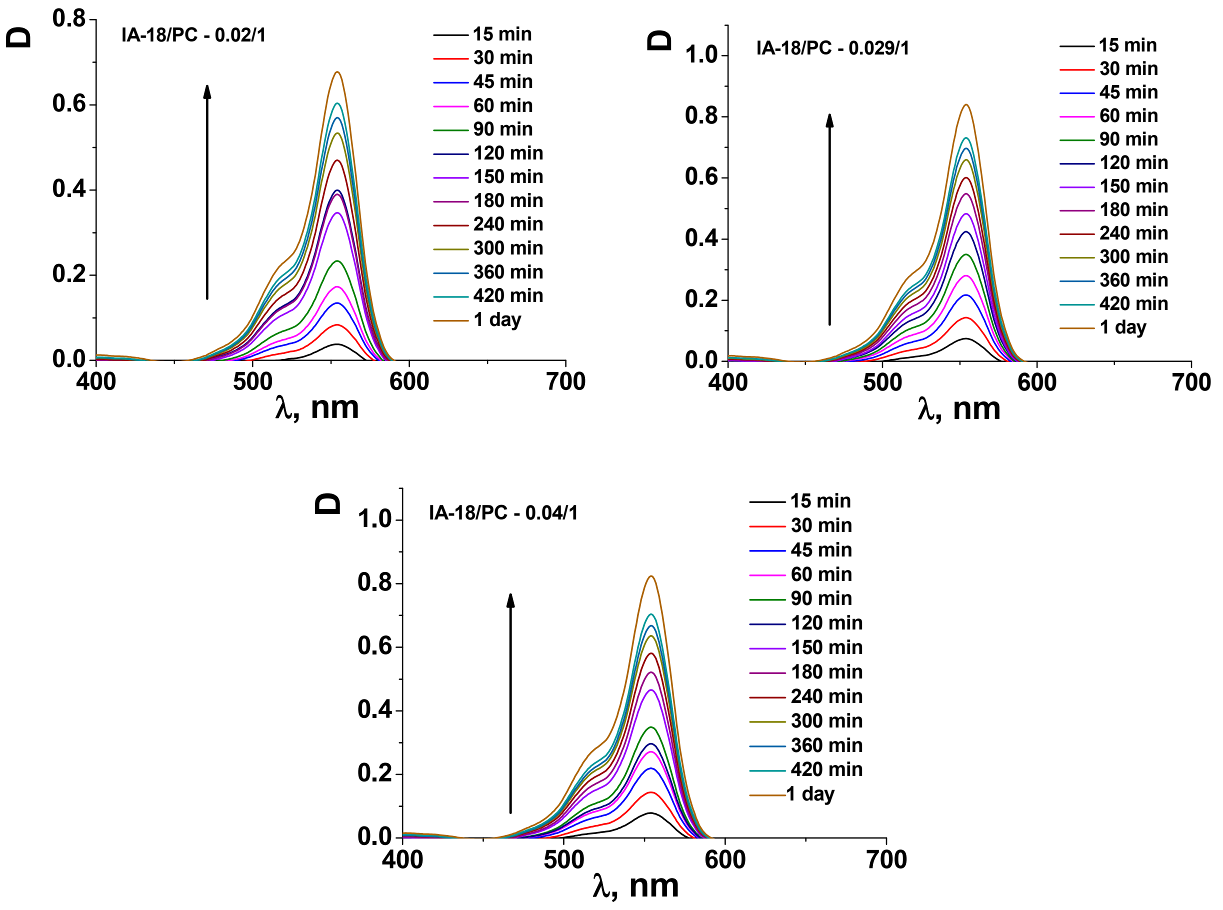

Figure S6. The absorption spectra of Rhodamine B at different release time intervals for modified IA-18/PC liposomes, molar ratio of components: 0.02/1; 0.029/1; 0.04/1; 37°C, phosphate buffer (0.025 M), pH = 7.4; the arrow indicates the direction of dialysis duration increasing.

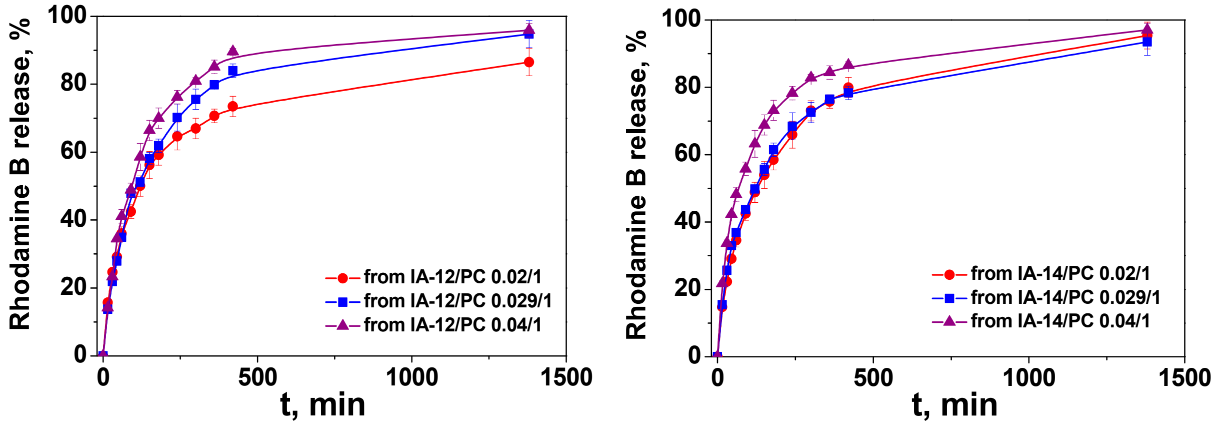

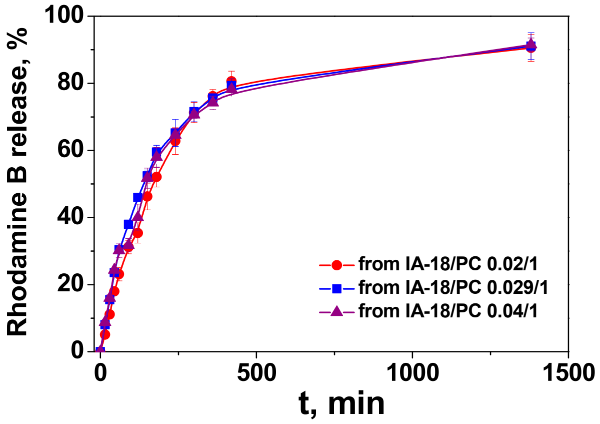

Figure S7. *In vitro* Rhodamine B release from mixed liposomes at various surfactant/lipid molar ratio: IA-12/PC; IA-14/PC; IA-18/PC; phosphate buffer (0.025 M), pH 7.4, 37°C.

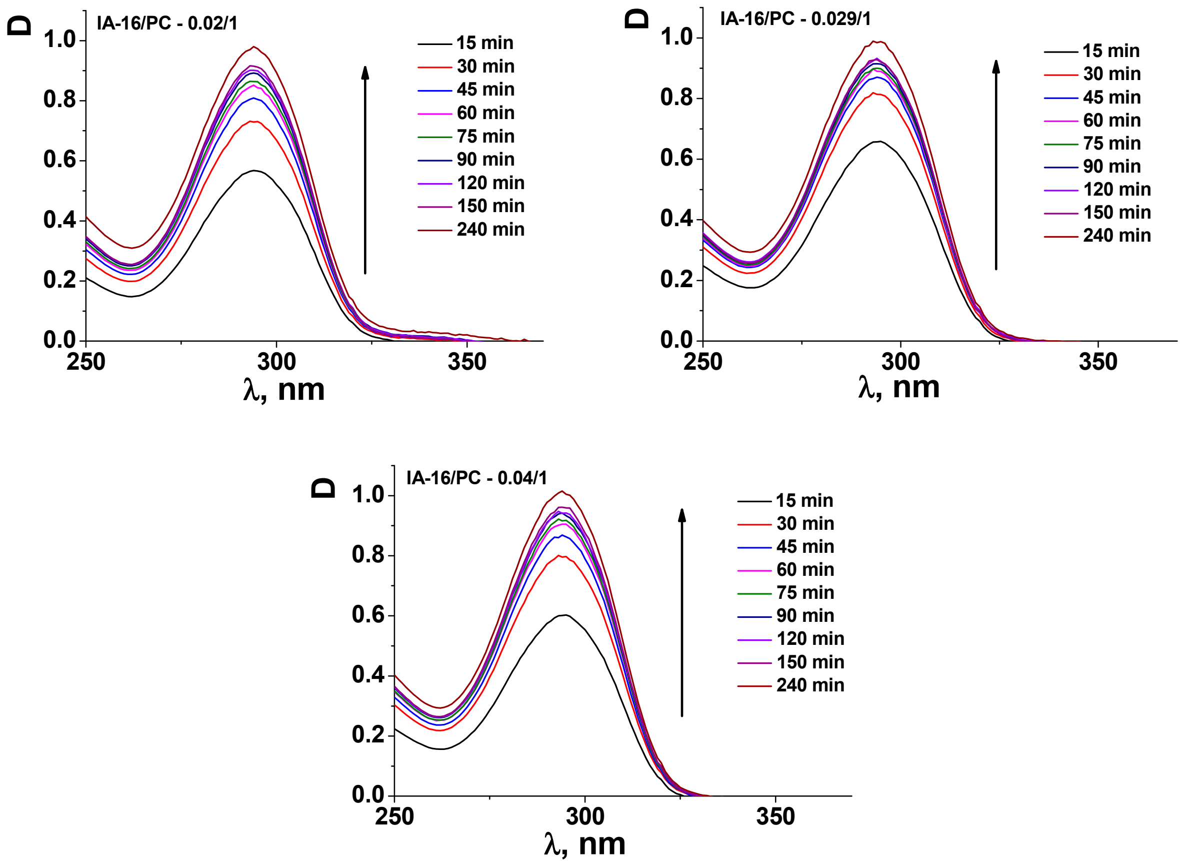

Figure S8. The absorption spectra of 2-PAM at different release time intervals for modified IA-16/PC liposomes, molar ratio of components: 0.02/1; 0.029/1; 0.04/1; 37°C, C (2-PAM) = 10 mg/mL; phosphate buffer (0.025 M), pH = 7.4; the arrow indicates the direction of dialysis duration increasing.

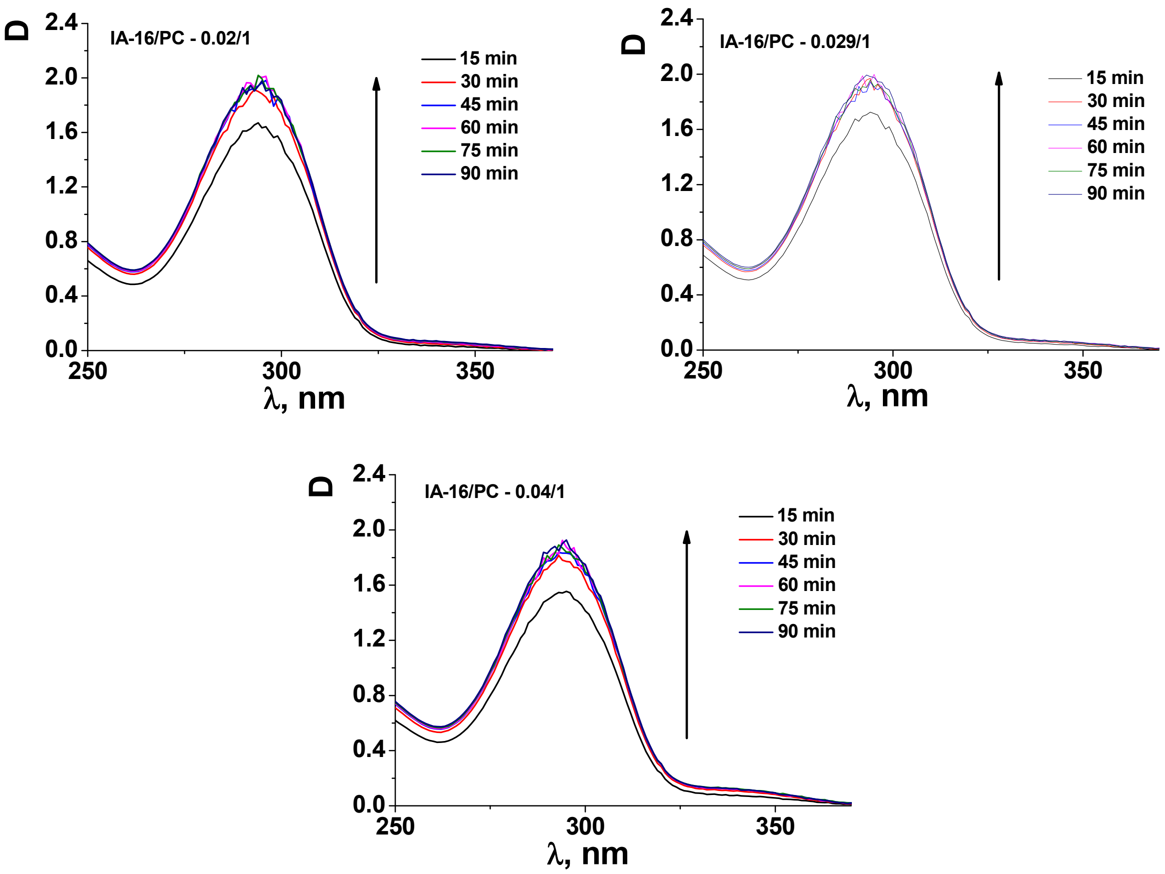

Figure S9. The absorption spectra of 2-PAM at different release time intervals for modified IA-16/PC liposomes, molar ratio of components: 0.02/1; 0.029/1; 0.04/1; 37°C, C (2-PAM) = 20 mg/mL; phosphate buffer (0.025 M), pH = 7.4; the arrow indicates the direction of dialysis duration increasing.

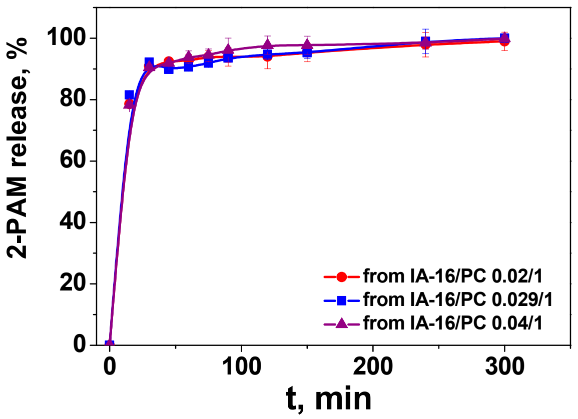

Figure S10. *In vitro* 2-PAM release from IA-16/PC modified liposomes using the dialysis bag method (n = 3); C (2-PAM) = 20 mg/mL, phosphate buffer (0.025 M), pH 7.4, 37°C.
